# The Effect of a Second Dose of Measles Vaccine at 18 Months of Age on Nonaccidental Deaths and Hospital Admissions in Guinea-Bissau: Interim Analysis of a Randomized Controlled Trial^[Author-notes ciac155-FM1]^

**DOI:** 10.1093/cid/ciac155

**Published:** 2022-02-26

**Authors:** Mike L T Berendsen, Isaquel Silva, Carlitos Balé, Sebastian Nielsen, Sophus Hvidt, Cesario L Martins, Christine S Benn, Peter Aaby

**Affiliations:** Bandim Health Project, Department of Clinical Research, University of Southern Denmark and Odense University Hospital, Odense, Denmark; Bandim Health Project, Indepth Network, Bissau, Guinea-Bissau; Department of Internal Medicine, Radboud Center for Infectious Diseases, Radboud University Medical Center, Nijmegen, The Netherlands; Bandim Health Project, Indepth Network, Bissau, Guinea-Bissau; Bandim Health Project, Indepth Network, Bissau, Guinea-Bissau; Bandim Health Project, Department of Clinical Research, University of Southern Denmark and Odense University Hospital, Odense, Denmark; Bandim Health Project, Indepth Network, Bissau, Guinea-Bissau; Bandim Health Project, Indepth Network, Bissau, Guinea-Bissau; Bandim Health Project, Indepth Network, Bissau, Guinea-Bissau; Bandim Health Project, Department of Clinical Research, University of Southern Denmark and Odense University Hospital, Odense, Denmark; Danish Institute for Advanced Study, University of Southern Denmark, Odense, Denmark; Bandim Health Project, Indepth Network, Bissau, Guinea-Bissau

**Keywords:** measles vaccine, booster dose, measles eradication, non-specific effects of vaccines, heterologous effects

## Abstract

**Background:**

The world is set on the eradication of measles. Continuation of the measles vaccine (MV) after eradication could still reduce morbidity because the MV has so-called beneficial nonspecific effects. We evaluated the effect of a “booster” dose of the MV on overall severe morbidity.

**Methods:**

We conducted a randomized controlled trial among children aged 17.5 to 48 months in Guinea-Bissau, where the MV is recommended only at 9 months of age. At the time of this interim analysis, 3164 children had been allocated 1:1 to a second dose of measles vaccine (MV2) at 18 months of age or to no vaccine. Severe morbidity (a composite outcome of nonaccidental deaths and hospital admissions) rate ratios (SMRRs) were calculated by Cox regression analysis censored for national oral polio vaccine (OPV) campaigns.

**Results:**

There were no measles cases during the trial period. There were 43 nonaccidental deaths or hospital admissions during follow-up. Severe morbidity was 2.6 per 100 person-years in the MV2 group and 3.6 per 100 person-years among controls; hence, the estimated effect of MV2 on severe morbidity was 28% (SMRR, 0.72; 95% confidence interval [CI], .38–1.38). At 12 months of follow-up, the number needed to treat to prevent 1 severe morbidity event was 137 children. After OPV campaigns, the estimated effect of MV2 was reduced to 9% (SMRR, 0.91; 95% CI, .46–1.81).

**Conclusions:**

MV2 may reduce nonmeasles severe morbidity by 28% (−38% to 62%), although this did not achieve statistical significance in this study. If significant in higher powered studies, this has major implications for child health, even after measles eradication.

**Clinical Trials Registration:**

NCT02943681.

## Teaser

A second dose of measles vaccine (MV) at 18 months of age tended to reduce hospital admissions and deaths by 28% (95% confidence interval [CI], −38%–62%); the estimate was 36% (95% CI, −28%–69%) after the adverse events window of MV.

In 2001, the Measles and Rubella Initiative was established to promote eradication of measles [1]. According to the World Health Organization (WHO), measles eradication is feasible, beneficial, and cost-effective [2–4]. Eradication has only been accomplished for smallpox [5]. Although theoretical, similar posteradication steps might be pursued for measles, with the phaseout of extra doses and ultimately cessation of the program. However, if vaccines have beneficial effects beyond their target-pathogen [6], these benefits might support continuing vaccination after eradication. Harnessing these beneficial nonspecific effects (NSEs) after eradication could still increase overall survival, as has been proposed for smallpox [7, 8].

There is increasing evidence that measles vaccine (MV) possesses NSEs. Studies of MV introduction in Africa reported reductions in mortality of 50%, which were higher than the expected prevention of ± 10% of measles-related deaths [9, 10]. These findings were supported by subsequent observational studies and randomized controlled trials (RCTs) [11]. Moreover, receiving a “booster” dose could increase the beneficial NSEs for several live-attenuated vaccines [12–17]. However, combining different live-attenuated vaccines might not result in additive beneficial effects, as seen for oral polio vaccine (OPV) and MV [18–20].

We aimed to study the effect of a second measles vaccine dose (MV2) at 18 months of age on nonaccidental mortality and hospital admissions in Guinea-Bissau. The recommended schedule included a single MV at 9 months of age. Because of the coronavirus disease 2019 epidemic and national plans to introduce MV2 in response to new WHO recommendations that all countries should introduce MV2, regardless of MV1 coverage levels [21], we will not be able to continue the trial and therefore decided that the planned interim analysis in 2019 would be the final report.

## METHODS

### Setting

The study took place at the Bandim Health Project (BHP) in Bissau, the capital of Guinea-Bissau. The BHP Health and Demographic Surveillance System (HDSS) registers vaccinations, admissions, infectious diseases, and survival for all children < 3 years of age at 4-monthly household visits. BHP also registers admissions, consultations, and vaccinations at the national hospital’s pediatric ward and at 3 HDSS health centers. The vaccination schedule consists of bacillus Calmette-Guérin (BCG) and OPV at birth; pentavalent vaccine (Penta), OPV, and pneumococcal conjugate vaccine (PCV) at 6, 10, and 14 weeks; rotavirus vaccine at 6 and 10 weeks; and MV1 and yellow fever vaccine at 9 months [22]. MV1 coverage was 71% in Guinea-Bissau in 2016, but 76% in the HDSS area [23].

During the trial, there was very little measles circulating in Guinea-Bissau [24]. All trial participants suspect of measles were tested at the national laboratory. As a result, samples of 2 participants with suspected measles were tested, but both were immunoglobulin M negative.

### Study Design

This RCT was designed to examine the effects of MV2 on “severe morbidity,” a composite outcome of nonaccidental mortality and hospital admissions, in children aged 17.5–48 months. The trial was initiated on 25 October 2016 and follow-up and enrollment continued up to the national MV campaign on 3 May 2019. Inclusion criteria were age 17.5–24 months and fulfilled vaccination criteria (received MV1, received all doses of the nonlive vaccines [Penta, PCV] before MV1, not yet received MV2). After IPV was added to the OPV3/Penta3/PCV3 vaccinations in January 2016, children should also have received IPV before MV1; however, IPV was not available between August 2017 and May 2018. Exclusion criteria were major malformations, overt illness, or participation in an early 2-dose MV trial. Children that received a nonlive meningococcal vaccine between MV1 and study enrollment during a national campaign in June 2016, targeting individuals aged 1–29 years, were also excluded from the analyses.

### Enrollment and Informed Consent

HDSS children that fulfilled the inclusion criteria were visited and invited to enroll at the health center. At the health centers, anthropometric data were collected, as were the child’s and its mother’s BCG scar status.

Mothers/guardians received written and oral explanation of the study. Provided oral consent, they signed the consent form by signature or fingerprint. In case of a fingerprint, a second person signed the document.

### Randomization Procedure

The children were randomized (1:1) in blocks of 24. Allocation concealment was ensured by using opaque envelopes. Because NSEs of MV might differ by sex [25], males and females were randomized separately. Same-sex twins were allocated to the same group. Children in the intervention group received a standard-titer Edmonston-Zagreb vaccine (Serum Institute of India). Mothers of control children were told that their child would receive a second dose at the end of the study. We did not provide a placebo/comparator vaccine.

### Sample Size

Based on previous experiences, we expected to recruit 8500 children over a 4-year period. With a mean follow-up of 25.4 months, a mortality and admission risk of 2.5% and 17.5%, respectively, and a loss to follow-up of 15%, this would result in a severe morbidity of 9.4/100 person-years (p-y) with a minimal detectable effect size of 15% reduction in severe morbidity (1-β = .8, α = .05). For the interim analysis, planned at the national MV campaign in 2019, we expected to have recruited 4750 children. With an assumed severe morbidity risk of 9% during a mean follow-up of 13.5 months, the severe morbidity would be 8/100 p-y with a minimal detectable effect size of 26% reduction. These effect sizes are within the range found in previous trials on NSEs of MV [18, 25, 26].

### Outcomes and Follow-up

The primary outcome was severe morbidity up to 48 months of age with sex-specific estimates as coprimary outcomes. By protocol, follow-up would be censored at the time of OPV campaigns (initiated on 24 November 2017 and 20 April 2018), but results supporting MV-OPV interactions made us conduct separate analyses for the time before and after OPV campaigns [18–20]. Children were not individually censored at national OPV campaigns because of a lack of individual-level data, but coverage of these campaigns has been >85% in this age group [27]. Distribution of follow-up time per child up to OPV campaigns is shown in Supplementary Figure 1. Deaths and admissions were detected through the 4-monthly HDSS visits, the 6-monthly study visits, and at the pediatric ward of the national hospital, where most children from BHP are admitted. Admissions were linked using a data linkage protocol (Supplementary Text). Reported admissions to smaller hospitals were not considered because they could not be verified due to absence of BHP registrars. For all deaths, a verbal autopsy was conducted with relatives by a trained field assistant. Consultations (ie, sick visits derived from health records) were a secondary outcome; the rate of consultations was used as a proxy for adverse reactions in the weeks after MV2.

### Statistical Analyses

Hazard ratios and Wald 95% confidence intervals (CIs) were estimated from an Andersen–Gill (A–G) model with time since enrollment as the underlying time. HRs were interpreted as severe morbidity risk ratios (SMRRs). For the analyses on deaths or admissions, these were interpreted as mortality or morbidity rate ratios, respectively. Follow-up started at enrollment and continued until censoring at migration, 48 months of age, or national OPV or MV campaign, whichever came first. Migrating children were censored at date of moving, if available, or date of last visit.

Admissions were analyzed as recurrent events, and admissions ≥1 day from the latest discharge counted as a new event. Admissions that resulted in death were counted only once in the severe morbidity outcome. We adjusted for dependency of same-sex twins and recurrent admissions using robust standard errors (clustering on identification/same-sex twins) and allowed different baseline hazards for males and females. Proportional hazard assumption was assessed visually and tested by Schoenfeld residuals, which revealed no nonproportionality.

Predefined effect modification analyses were performed for season (enrollment and at risk) because morbidity and mortality is often higher during the rainy season (dry season: 1 Dec–31 May; rainy season: 1 Jun–30 Nov). For season at risk, the follow-up time was distributed over these categories. We added interaction terms to the A-G model to compare strata of season and OPV campaign status, adjusting for dependence, as done in the main analyses.

MV causes mild adverse events that manifest within 2 weeks [21, 28]. For the adverse events analysis, the combined consultation rate at the national hospital and the 3 HDSS health centers in the first 14 days, was estimated using a similar A–G model as for severe morbidity.

We calculated a number needed to treat to benefit/harm (NNTB/NNTH) following Altman and Andersen (Supplementary Text) [29].

All analyses were 2-sided using Stata MP 13, StataCorp LLC, with 0.05 as the level for statistical significance.

### Ethics

The Guinean Ministry of Health’s Research Coordination Committee approved this RCT with consultative approval from the Danish Central Ethical Committee. The trial was registered at clinicaltrials.gov (NCT02943681). Further ethical considerations and MV2 receipt of controls is described in Supplementary Text.

## RESULTS

**Figure 1.**
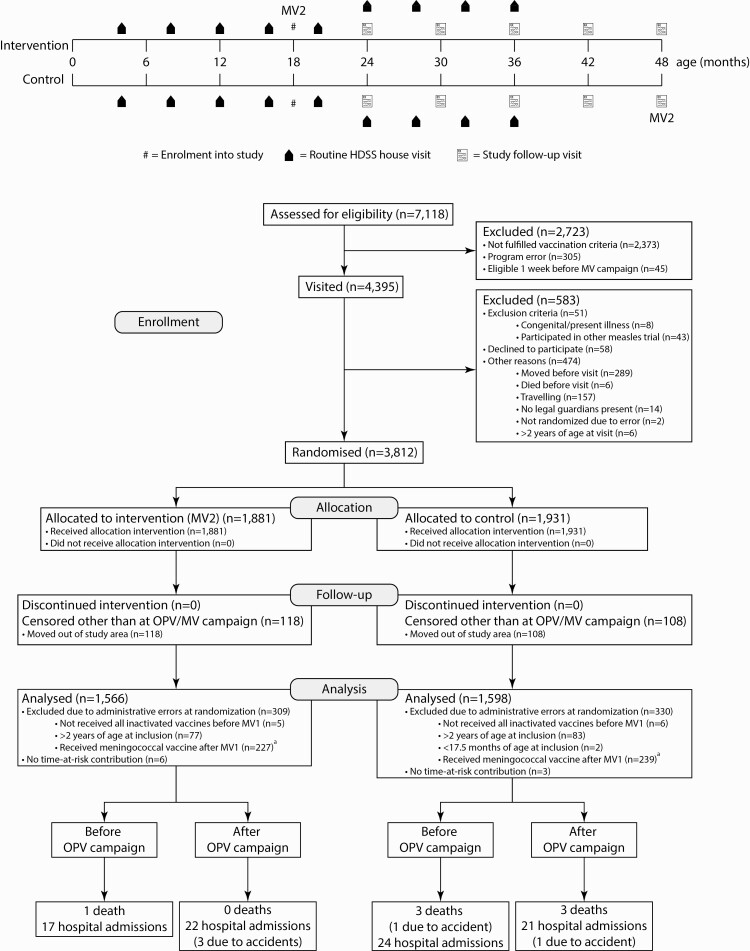
**Study design and flowchart according to the CONSORT guidelines.** In the control group, before any OPV campaign, 1 death was in-hospital and is therefore only counted as a hospital admission for severe morbidity and 1 death was due to an accident, resulting in a combined nonaccidental severe morbidity count of 25 (1 death + 24 hospital admissions). After OPV campaigns in that group, 2 deaths were in-hospital and are only counted as hospital admissions for severe morbidity and 1 hospital admission was due to an accident, resulting in a combined nonaccidental severe morbidity count of 21 (1 death + 20 hospital admissions). ^a^Children that had received the meningococcal vaccine between MV1 and study enrollment at the national campaign in 2016 were excluded from the analyses as they had received a nonlive vaccine after their MV1. Abbreviations: HDSS, Health and Demographic Surveillance System; MV1, first measles vaccine; MV2, second measles vaccine; OPV, oral polio vaccine.

During the trial, 7118 children were assessed for eligibility, of whom 2723 were excluded because they had not received MV1 and Penta + PCV3 (n = 1860; 645 missed MV1, 287 missed Penta3 and/or PCV3 and 928 missed MV1 and 1 of the nonlive vaccines), had received MV2 elsewhere (n = 4), had received a nonlive vaccine after MV1 (n = 509), had a program error (n = 305), or there was insufficient time for a house visit (n = 45). Among those visited, another 583 were excluded because they fulfilled an exclusion criterion (n = 51), declined participation (n = 58), had moved (n = 289) or died (n = 6), were travelling (n = 157), or had another reason (n = 22). Of the remaining 3812 children, 1881 received MV2 and 1931 were controls. Six-hundred and forty-eight randomized children (17%) were excluded from the analyses for several reasons (Figure 1). Demographics of excluded children are displayed in Supplementary Table 1. Baseline characteristics of the remaining 3164 children (1566 MV2, 1598 control) differed only in that MV2 recipients had a lower weight (10.12 kg vs 10.25 kg, *P* = .02) and lower maternal mid-upper arm circumference (268 mm vs 274 mm, *P* = .005) than controls (Table 1, missing in Supplementary Table 2). Censoring, other than at the national OPV/MV campaign, occurred for 226 (MV2, 118; control, 108) children who had moved out of the study area.

**Table 1.** Baseline Characteristics by MV2 Allocation

Characteristic	MV2 (n = 1566)	Control (n = 1598)
**Child factors**		
Male sex	50% (783)	50% (805)
Age, mo	17.6 (17.6–18.1)	17.7 (17.6–18.1)
Anthropometrics at enrollment		
Weight, kg^a^	10.12 (9.30–11.00)	10.25 (9.40–11.15)
Height, cm^a^	79.4 (77.2–81.5)	79.7 (77.5–81.7)
MUAC, mm^a^	142 (136–154)	148 (136–154)
Temperature, °C^a^	36.3 (36.0–36.6)	36.4 (36.0–36.6)
BCG scar^a^	90.8% (1419)	91.6% (1458)
Reported symptoms week before enrollment		
Any symptom^a^	58% (901)	59% (947)
Congestion/rhinorrhea^a^	41% (638)	41% (660)
Cough^a^	29% (461)	28% (442)
Fever^a^	22% (349)	24% (388)
Vomiting^a^	2.1% (33)	2.9% (46)
Diarrhea^a^	9.1% (142)	10% (164)
Rattle/wheeze^a^	4.4% (68)	3.1% (49)
Convulsions^a^	0	0.1% (1)
Other symptoms	6.8% (107)	5.9% (95)
Reported medication use week before enrollment	32% (495)	34% (542)
Paracetamol^a^	27% (428)	30% (481)
Antimalarials^a^	0.02% (29)	0.01% (21)
Antibiotics^a^	17% (271)	19% (310)
Still breastfed^a^	68% (1057)	67% (1074)
Hospital admission before inclusion^a^	14% (217)	12% (185)
Child has had measles^a,b^	0.2% (3)	0.2% (3)
**Socioeconomic factors**		
Zinc roofing material^a^	99% (1539)	99% (1573)
Functioning electricity in home^a^	34% (531)	33% (517)
Indoor toilet^a^	26% (410)	29% (452)
Number of persons per room^a^	4 (3–5)	4 (3–5)
Number of persons per bed^a^	2 (2–3)	2 (2–3)
Sleeping under bed net^a^	99% (1537)	99% (1565)
Telephone available	91% (1426)	92% (1478)
Antimalarials in the household^a^	0.2% (3)	0.5% (8)
Pigs in the household^a^	17% (270)	15% (242)
Measles in the household^a,b^	0.1% (1)	0.3% (4)
**Maternal factors**		
Mother not alive	0.3% (5)	0.2% (3)
Maternal age, y^a^	28 (23–32)	28 (24–32)
Maternal schooling^a^	81% (1231)	80% (1243)
Years of schooling^a^	9 (7–12)	9 (6–12)
Maternal MUAC^a^	268 (250–298)	274 (250–304)
Maternal measles infection^a^	11% (161)	13% (186)
Maternal measles vaccination^a^	99% (1472)	98% (1479)
Maternal BCG scar^a^	69% (966)	68% (976)

Results are presented as median (25th percentile–75th percentile) for continuous variables and as percentages (n) for categorical variables.

Abbreviations: BCG, bacillus Calmette-Guérin; MUAC, mid-upper arm circumference; MV2, second dose of measles vaccine.

Different n because of missing values (numbers in supplementary Table 5).

Measles cases were reported by mother or other relative of the child and could not be confirmed in the routine data collection of the Health and Demographic Surveillance System. We assume these cases to be different childhood infections that might have had similar characteristics, such as varicella.

### Severe Morbidity After MV2

There were 83 nonaccidental deaths or admissions between inclusion and 48 months of age (Figure 1). Therefore, the actual severe morbidity (3.1/100 p-y) was almost 3 times lower than the expected severe morbidity (8.0/100 p-y). No death or admission was caused by measles infection.

**Figure 2.**
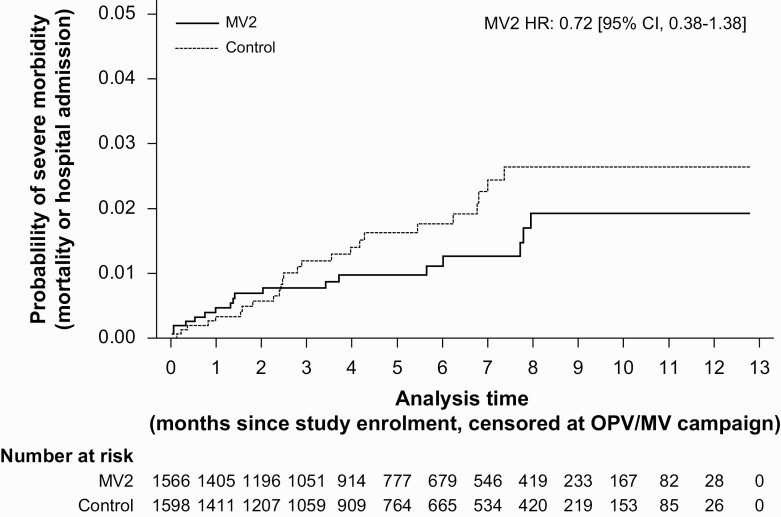
**Kaplan-Meier curve of severe morbidity probability by MV2 allocation.** Severe morbidity rate ratio (SMRR) was estimated from a Cox proportional hazards model with time since enrollment as the underlying time variable and observations were censored at migration, 48 months of age or first day of national OPV or MV campaign. Hospital admissions were analyzed as recurrent events, and hospital admissions ≥1 day from the latest discharge counted as a new event. Hospital admissions that resulted in death were counted as a singular event. Abbreviations: MV2, second measles vaccine; OPV, oral polio vaccine.

**Figure 3.**
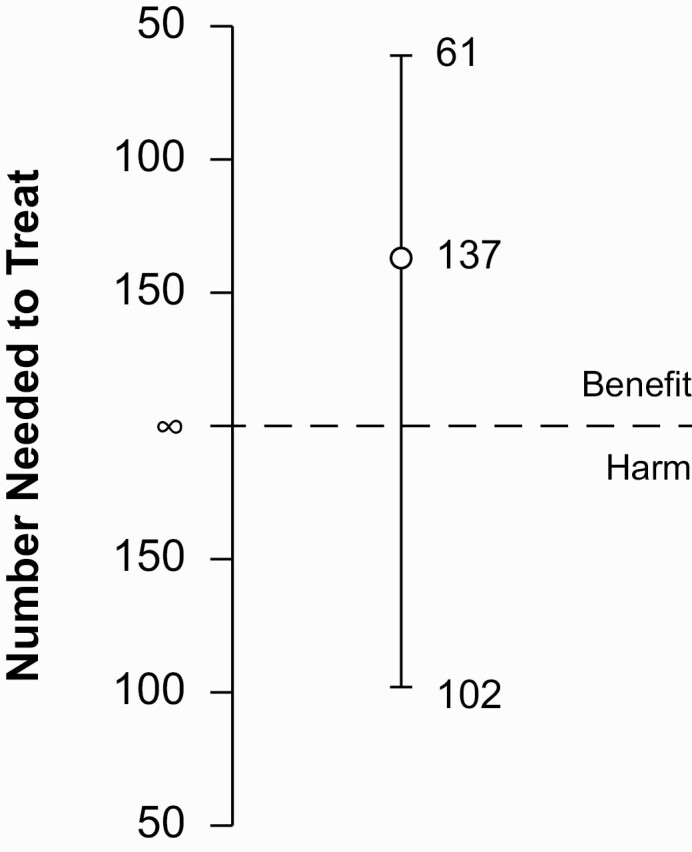
**Graphical depiction of the NNTB/NNTH confidence interval.** In contrast to the severe morbidity risk ratio (SMRR), the number needed to treat (NNT) does not shift from beneficial (NNTB) to harmful (NNTH) at a value of one, but rather at infinity. This is because at an SMRR of 1, it would require vaccination of an infinite number of infants to prevent 1 severe morbidity event. With our numbers, it means that we would need to vaccinate 137 children to prevent 1 event. The 95% confidence interval shows that this can be as few as only 61 children needed to be vaccinated to prevent 1 event. The other side of the 95% confidence interval indicates that we could also cause 1 event by vaccinating 102 children.

Forty-three events occurred before OPV campaigns. Severe morbidity was lower among MV2 recipients (2.6/100 p-y) than controls (3.6/100 p-y), the SMRR being 0.72 (95% CI, .38–1.38) (Table 2, Figure 2). The resulting NNTB at 12 months of follow-up was 137 (95% CI, NNTH 102 to ∞ to NNTB 61, Figure 3), meaning that 137 children had to receive MV2 to prevent 1 event.

**Table 2.** Severe Morbidity by MV2 Allocation, Overall and Sensitivity Analysis With Follow-up Time Split at Different Points

	Severe Morbidity Rate [Deaths or Hospital Admissions/100 Person-years] (n)		SMRR (95% CI) (MV2/control)
	MV2	control	
Complete follow-up period	2.6 [18/6.9] (1566)	3.6 [25/6.9] (1598)	0.72 (.38–1.38)
**Split at 14 days**			
First 14 d after inclusion	6.8 [4/0.6] (1566)	5.0 [3/0.6] (1598)	1.36 (.30–6.08)^a^
>14 ds after inclusion	2.2 [14/6.3] (1479)	3.5 [22/6.3] (1505)	0.64 (.31–1.28)^a^
**Split at 21 d**			
First 21 d after inclusion	5.8 [5/0.9] (1566)	3.4 [3/0.9] (1598)	1.70 (.40–7.12)^b^
>21 d after inclusion	2.2 [13/6.0] (1441)	3.7 [22/6.0] (1449)	0.59 (.29–1.21)^b^

SMRRs were estimated from Cox proportional hazards models with time since inclusion as the underlying time variable and observations were censored at migration, 48 months of age or first day of national OPV or MV campaign. Hospital admissions were analyzed as recurrent events, and hospital admissions ≥1 day from the latest discharge counted as a new event. Hospital admissions that resulted in death were counted as a singular event.

Abbreviations: CI, confidence interval; MV, measles vaccine; MV2, second measles vaccine; OPV, oral polio vaccine; SMRR, severe morbidity rate ratio.

Proportional hazards test, *P* = 1.00.

Proportional hazards test, *P* = .97.

A visible difference in the hazards during follow-up, coinciding with the known window of possible adverse events, made us analyze the data by period of follow-up. During the first 14 days, the SMRR was 1.36 (95% CI, .30–6.08), whereas this was 0.64 (95% CI, .31–1.28) after 14 days (*P* for interaction = .35) (Table 2, Supplementary Figure 2). Extending this window to 21 days in a sensitivity analysis, the SMRRs were 1.70 (95% CI, .40–7.12) during and 0.59 (95% CI, .29–1.21) after the window (*P* for interaction = .18) (Table 2).

By sex, the overall SMRR was 0.78 (95% CI, .34–1.82) for males and 0.66 (95% CI, .24–1.79) for females (*P* for interaction = .80) (Table 3, Supplementary Figure 3).

**Table 3.** Severe Morbidity by MV2 Allocation, Overall and by Sex

	Severe Morbidity Rate [Deaths or Hospital Admissions/100 Person-years] (n)		SMRR (95% CI) (MV2/control)
	MV2	control	
**Overall**			
Complete follow-up period	2.6 [18/6.9] (1566)	3.6 [25/6.9] (1598)	0.72 (.38–1.38)^a^
**Males**			
Complete follow-up period	2.9 [10/3.5] (783)	3.7 [13/3.5] (805)	0.78 (.34–1.82)^b^
**Females**			
Complete follow-up period	2.3 [8/3.4] (783)	3.6 [12/3.4] (793)	0.66 (.24–1.79)^b^

SMRRs were estimated from Cox proportional hazards models with time since inclusion as the underlying time variable and observations were censored at migration, 48 months of age, or first day of national OPV or MV campaign. Hospital admissions were analysed as recurrent events, and hospital admissions ≥1 day from the latest discharge counted as a new event. Hospital admissions that resulted in death were counted as a singular event.

Abbreviations: CI, confidence interval; MV, measles vaccine; MV2, second measles vaccine; OPV, oral polio vaccine; SMRR, severe morbidity rate ratio.

Proportional hazards test, *P* = .58.

Proportional hazards test, *P* = .37.

### Mortality and Hospital Admissions

Severe morbidity, split into its separate components (deaths and admissions), revealed an overall mortality of 0.2/100 p-y and an admission rate of 3.0/100 p-y, the HRs for MV2 being 0.50 (95% CI, .04–5.46) for mortality and 0.71 (95% CI, .36–1.38) for admissions (Supplementary Table 3).

### Influence of OPV Campaigns

**Figure 4.**
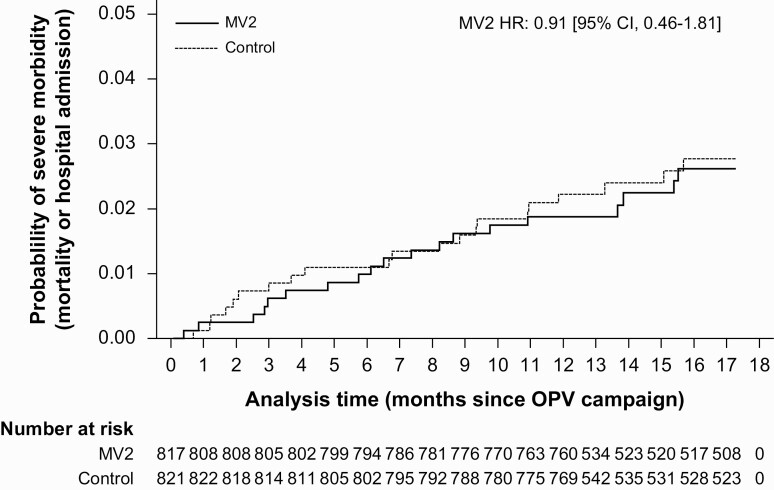
**Kaplan-Meier curve of severe morbidity probability by MV2 allocation, after OPV campaign.** Severe morbidity rate ratio (SMRR) was estimated from a Cox proportional hazards model with time since OPV campaign as the underlying time variable and observations were censored at migration, 48 months of age, or first day of national MV campaign. Hospital admissions were analyzed as recurrent events, and hospital admissions ≥1 day from the latest discharge counted as a new event. Hospital admissions that resulted in death were counted as a singular event. Proportional hazards test, *P* = .92. Abbreviations: MV2, second measles vaccine; OPV, oral polio vaccine.

After OPV campaigns, there were 40 events. Severe morbidity rates were 1.8/100 p-y among MV2 recipients and 2.0/100 p-y among controls, the SMRR being 0.91 (95% CI, .46–1.81; *P* for interaction = .62) (Figure 4). The Kaplan-Meier curve without censoring at OPV campaigns is shown in Supplementary Figure 4.

### Seasonal Differences

Enrollment in the dry season resulted in an SMRR of 0.85 (95% CI, .31–2.34), whereas the SMRR was 0.66 (95% CI, .29–1.53) for rainy season enrolment (*P* for interaction = .70). A similar trend was seen for season at risk; 0.91 (95% CI, .37–2.22) during the dry season and 0.59 (95% CI, .25–1.41) during the rainy season (*P* for interaction = .48) (Supplementary Table 4, Supplementary Figure 5).

### Minor Adverse Events

In the first 14 days, there was no difference in consultation rates between the MV2 group (185.7/100 p-y) and controls (186.6/100 p-y), the HR being 1.00 (95% CI, .76–1.30) (Supplementary Table 5).

## DISCUSSION

MV2 reduced nonaccidental deaths and admissions before OPV campaigns by an estimated 28% (95% CI, −38–62), although it did not reach statistical significance. A post hoc analysis showed that in the first 14 days, when adverse events of MV typically arise, the estimate was a 36% (95% CI, −70–508) increased risk of severe morbidity in the MV2 recipients, whereas after 14 days the estimate was a 36% (95% CI, −28–69) reduced risk. However, neither the estimates nor the interaction term reached statistical significance. There was no difference in consultation rate in the first 14 days.

Several reports on live vaccines have demonstrated that a “booster” dose increases their beneficial NSEs [12–17, 25]. Our results find a possibly similar effect. However, the smaller number of children included, a lower-than-expected mortality and admission rate, and OPV campaigns limiting the follow-up period, decreased our power of finding statistically significant results. Especially the small number of deaths has affected our statistical power for the mortality outcome. The lower mortality and admission rates were partly the result of improved hospital triage and care by Doctors Without Borders who supported the national hospital from February 2016 [30]. Our findings should be interpreted with caution. Nevertheless, there are several suggestions that this might not be merely a chance finding. First, the estimated effect is well within the range of previous studies on NSEs of MV [18, 25, 26]. Second, both mortality and admissions show concordant estimated effects of MV2. Third, the reduction in severe morbidity by MV2 was less pronounced after children participated in OPV campaigns; this effect modification is in line with previous findings that the effect of MV is decreased when the control group received OPV campaigns [18–20].

### Strengths and Weaknesses

We included only admissions registered at the pediatric ward of the national hospital because mothers/family members often have problems recalling time and nature of hospital visits and do not get any form of record. BHP does not record admissions in other hospitals and as such we could not verify or include the 109 reported admissions (MV2, 42; controls, 67) to those hospitals. This might have hampered the power of the study.

We chose not to use a placebo because this might have led the mothers to believe their child was already vaccinated and not seek vaccination should an MV2 policy be implemented. The use of comparator vaccines is also questionable; comparator vaccines can have NSEs that influence the outcome [31]. However, healthcare workers and field assistants responsible for follow-up were not aware of the randomization status.

Our vaccination inclusion criteria represent the WHO-recommended schedule and ensured no catchup vaccination of routine nonlive vaccines after enrollment. Nevertheless, it may limit the generalizability of our findings to a group of children that deviated from this schedule. The exclusion of additional children after randomization and censoring at migration could have led to selection bias. Although comparable characteristics of children excluded postrandomization and a similar number of children lost to follow-up (χ^2^*P* = .89) in the randomization groups provide some reassurance, selection bias cannot be ruled out.

### Comparison With Previous Studies

In previous MV trials, females benefited more from vaccination than males [25, 32, 33]. However, in those trials, the most recent vaccine among controls was a nonlive vaccine, associated with negative effects among females [31, 34]. This was clear in a recent trial of MV given before 9 months of age where females benefited more from MV before 9 months of age, whereas there were no significant sex-differential effects of MV after 9 months of age [26, 33].

Several studies have now reported beneficial NSEs after OPV [13, 17, 35] and modifying effects of OPV campaigns on the NSEs of other vaccines [15, 18–20]. The reduced MV2 estimate after OPV campaigns corroborate these findings and indicates that the relationship between NSEs of different live-attenuated vaccines is probably more complex than simple addition of effects.

## IMPLICATIONS AND CONCLUSION

The comparable consultation rates in both groups during the first 14 days indicates the safety of implementing MV2 [21]. Although admission rate in the MV2 group was slightly higher in this period, only 1 of 4 admissions was a classic adverse MV event: a febrile seizure. The other admissions were diagnosed as pneumonia + malnutrition, diarrheal disease + malnutrition, and gastroenteritis.

Next to the numerous studies describing NSEs of vaccines given in infancy [11, 25, 35, 36], this study suggests that NSEs of vaccines can affect child health when given during the second year of life. It also supports rethinking of posteradication planning. Continuation of MV after disease eradication might still improve child health by harnessing its NSEs [7]. However, although this RCT provides suggestions for the importance of “booster” doses of live vaccines to extend beneficial NSEs toward the 5 years of age mark, adequately powered studies are needed to provide conclusive evidence.

New studies might have a different design due to ethical considerations. With the 2017 WHO guidelines [21], MV2 became standard policy irrespective of MV1 coverage; not providing this vaccine will be deemed unethical. New studies on MV2 would, therefore, be of observational origin. Although this is often seen as suboptimal, large observational studies with rigorous statistical methods such as performed by Sørup [37] or Bardenheier [38] might still provide useful evidence. Any RCT on NSEs of vaccines will be done in an evolving context of vaccination policy and intermittent campaigns. This has to be acknowledged and, if possible, taken into account during the design and analysis of such trials.

In conclusion, a second dose of MV at 18 months of age showed an estimated reduction in nonaccidental deaths and admissions of 28% (95% CI, −38–62), although this did not achieve statistical significance. If confirmed in an appropriate powered study, this has major implications for child health, now and after measles eradication.

## Supplementary Data

Supplementary materials are available at *Clinical Infectious Diseases* online. Consisting of data provided by the authors to benefit the reader, the posted materials are not copyedited and are the sole responsibility of the authors, so questions or comments should be addressed to the corresponding author.

ciac155_suppl_Supplementary_Appendix

## Notes

***Author contributions.*** C. L. M., C. S. B., and P. A. conceived and designed the second dose of measles vaccine (MV) trial; C. S. B. and P. A. are the guarantors of the study. M. L. T. B., I. S., and S. H. supervised the field data collection of the trial. M. L. T. B. performed statistical analysis, assisted by S. N. and S. H. C. S. B. and P. A. critically interpreted the data. M. L. T. B. wrote the first draft of the manuscript. All authors contributed to the final version of the manuscript. All authors had full access to all of the data and take responsibility for the integrity of the data and the accuracy of the data analysis.

***Acknowledgments.*** The authors thank all infants and their guardians that participated in the study.

***Disclaimer.*** The funding agencies had no role in the study design, data collection, data analysis, data interpretation, or writing of the manuscript.

***Financial support.*** This work was supported by the Danish National Research Foundation (grant DNRF108 to the Research Center for Vitamins & Vaccines); the European Research Council (ERC Starting grant [#243149] to C. S. B.; the Novo Nordisk Foundation (research professorship grant to P. A.), and the University of Southern Denmark (SDU faculty scholarship, which funded M. L. T. B.).
