# Supplementary material for: The Effect of a Second Dose of Measles Vaccine at 18 Months of Age on Nonaccidental Deaths and Hospital Admissions in Guinea-Bissau: Interim Analysis of a Randomized Controlled Trial
Source: Clin Infect Dis. 2022 Feb 26;75(8):1370–8. doi: 10.1093/cid/ciac155 (PMC9797043; doi:10.1093/cid/ciac155)
Supplement: ciac155_suppl_Supplementary_Appendix [file ciac155_suppl_supplementary_appendix.docx]

**APPENDIX**

**Supplementary Text.** Systematic search for study infants in the paediatric ward database

**Supplementary Text.** Calculation of Number Needed to Treat to Benefit/Harm

**Supplementary Text.** Ethical considerations and MV2 receipt of controls

**Supplementary Figure 1.** Distribution of follow-up time for study participants

**Supplementary Figure 2.** Kaplan-Meier curve of severe morbidity probability by MV2 allocation, split at 14 days of follow-up

**Supplementary Figure 3.** Kaplan-Meier curve of sex-differential effects on severe morbidity probability by MV2 allocation

**Supplementary Figure 4.** Kaplan-Meier curve of severe morbidity probability by MV2 allocation not censoring at OPV campaigns

**Supplementary Figure 5.** Kaplan-Meier curve of seasonal influences on severe morbidity probability by MV2 allocation

**Supplementary Table 1.** Characteristics of included and excluded children

**Supplementary Table 2.** Number of missing values in baseline characteristics

**Supplementary Table 3.** Mortality and hospital admissions by MV2 allocation

**Supplementary Table 4.** Seasonal influences on severe morbidity by MV2 allocation

**Supplementary Table 5.** Consultations in first 14 days by MV2 allocation

**Systematic search for study infants in the paediatric ward database**

Between 08.00-20.00 during the week and between 08.00-15.30 during weekends, a trained BHP assistant registered all infants coming to the paediatric ward’s triage room by parental names, infant birth date or approximate age, sex, neighbourhood, family telephone numbers, date of entry, whether the child had been born at the hospital, study ID number and BHP HDSS ID number, if applicable. A BHP supervisor systematically identified all HDSS infants to apply the HDSS ID. If an infant known to be admitted could not be located, and families in neighbouring beds or treating physicians could not inform whether the infant had been discharged alive or not, the BHP paediatric ward data management supervisor telephoned the family to ensure outcome registration. To identify study infants with no study number recorded (due to having lost/exchanged vaccination card or if the mother had left the card at home), we applied the following systematic data search and linkage protocol:

1. Linking by unique study ID and/or HDSS ID number
2. Linking on all available telephone numbers recorded admission to the ward and at study enrolment.
3. Linking on date of birth of the child, for all children admitted during the follow-up period of the trial.
4. Linking on name of the child, for all children admitted during the follow-up period of the trial.
5. Upon following the above linking steps, data for each study participant registered to have been hospitalized was individually reviewed to verify the comparability between enrolment and paediatric ward data, by manual comparison of the following data points in the 2 databases: mother’s name, child’s name and sex, residential neighbourhood, birthdate and collected telephone numbers (if provided).

**Calculation of Number Needed to Treat to Benefit/Harm**

The number needed to treat to benefit/harm (NNTB/NNTH) was calculated using the formula 1/[S_control_(t)^SMRR^-S_control_(t)]. S_control_(t) is the Kaplan-Meier estimate in the control group and SMRR is the severe morbidity rate ratio for MV2 from the Andersen-Gill model.

**Ethical considerations and MV2 receipt of controls**

A Data Safety and Monitoring Board was established, and all invited children were offered free healthcare consultations and essential drugs. At trial initiation, MV1 coverage in Guinea-Bissau was below the 80% required by the WHO to implement MV2 as standard policy at that time. This policy was revised by the WHO after trial initiation in 2017, with removal of the 80% MV1 coverage requirement. Therefore, participation did not result in less children receiving MV2, especially as study children also participated in the national campaigns. As we provided MV2 for half of the children, the net result was more children being vaccinated. All randomized children were eligible to receive MV2. Of those that were randomized to the control group, 73% (n=1,404) received MV2 during the national campaign and 10% (n=200) had moved before the end of the study. For the other 17% (n=327), we lacked information on whether they had received MV2 during the national campaign as they were absent or travelling. However, none of these children had received their final study visit at the time of the national measles campaign.


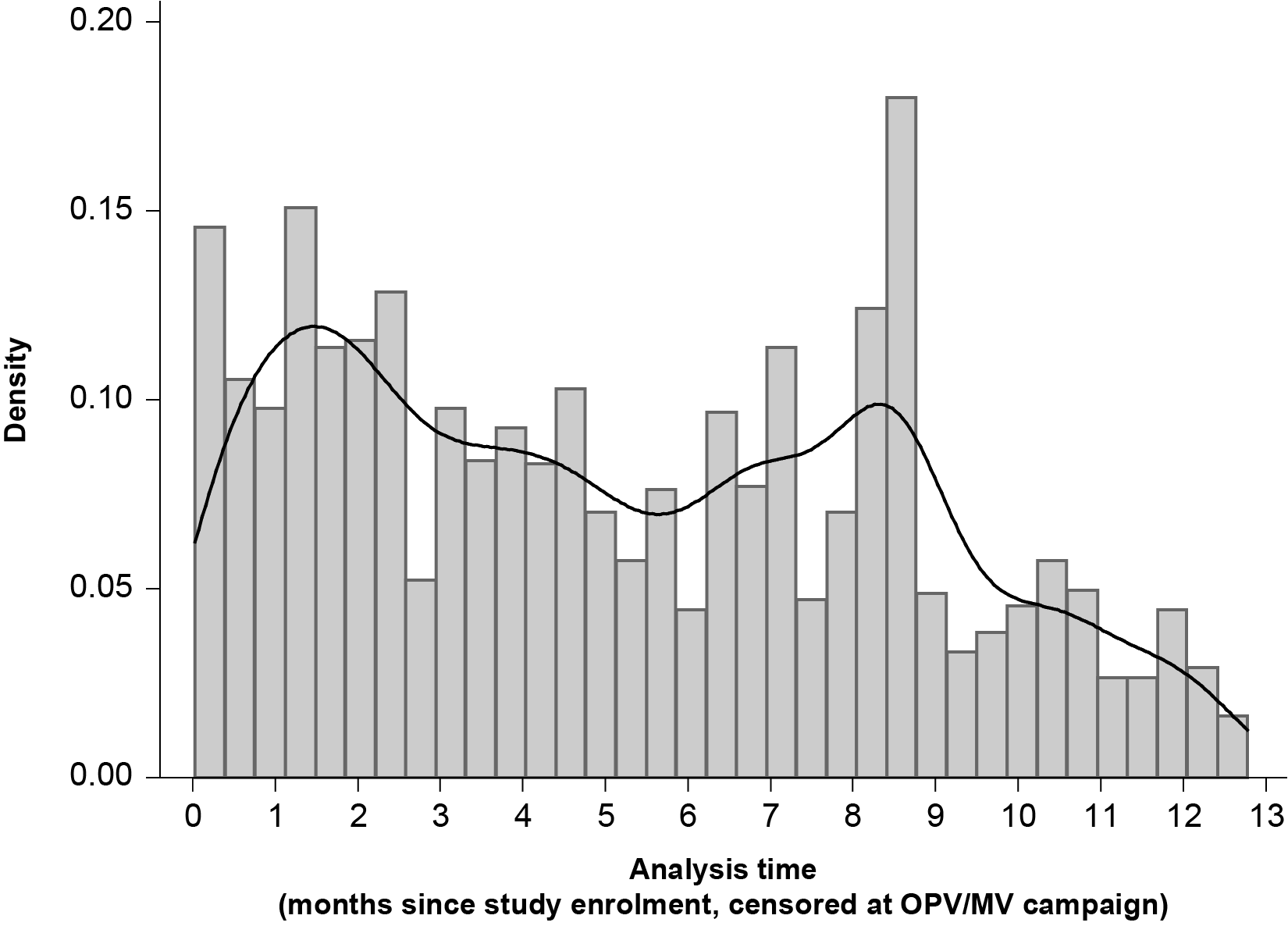


**Supplementary Figure 1. Distribution of follow-up time for study participants.** The graph depicts the kernel-density of the distribution of follow-up time for each individual study participant. Follow-up time was censored at migration, 48 months of age or first day of national OPV or MV campaign.


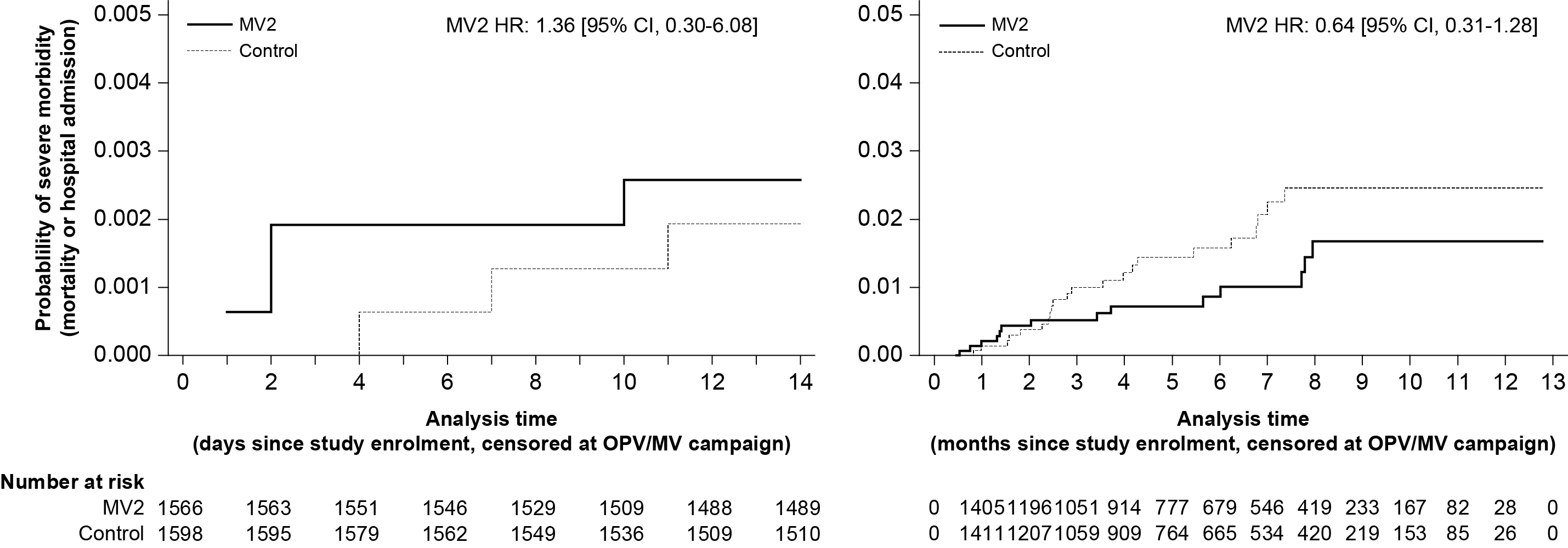


**Supplementary Figure 2. Kaplan-Meier Curve of severe morbidity probability by MV2 allocation split at 14 days of follow-up.** Severe morbidity rate ratios (SMRRs) for the first 14 days (A) and after the 14th day (B) were estimated from Cox proportional hazards models with time since enrolment as the underlying time variable and observations were censored at migration, 48 months of age or first day of national OPV or MV campaign. Hospital admissions were analysed as recurrent events, and hospital admissions >=1 day from the latest discharge counted as a new event. Hospital admissions that resulted in death were counted as a singular event. Note the differences in the axis. Abbreviations: MV2, Second measles vaccine.


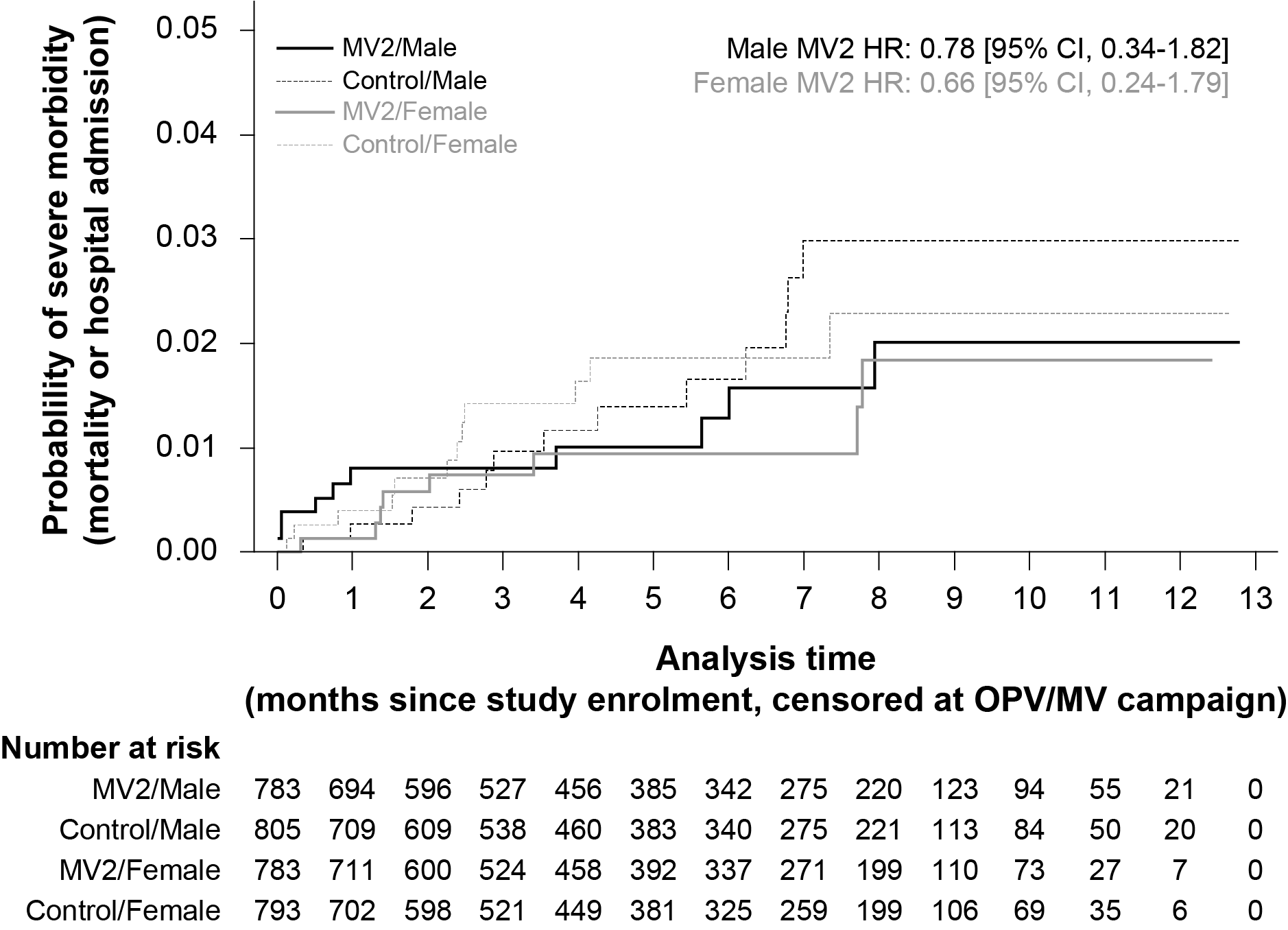


**Supplementary Figure 3. Kaplan-Meier curve of sex-differential effects on severe morbidity probability by MV2 allocation.** Severe morbidity rate ratio (SMRR) was estimated from a Cox proportional hazards model including an interaction term between allocation and sex with time since enrolment as the underlying time variable and observations were censored at migration, 48 months of age or first day of national OPV or MV campaign. Hospital admissions were analysed as recurrent events, and hospital admissions >=1 day from the latest discharge counted as a new event. Hospital admissions that resulted in death were counted as a singular event. Abbreviations: MV2, Second measles vaccine.


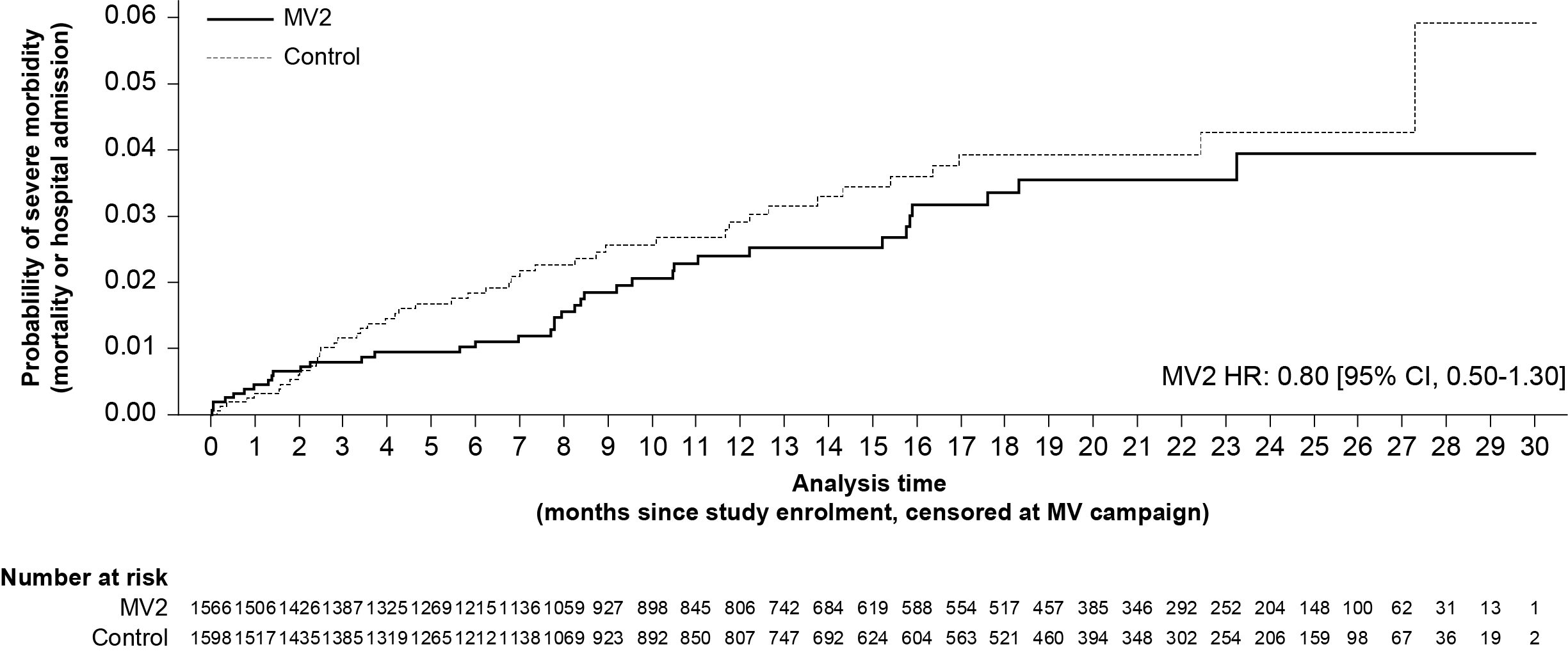


**Supplementary Figure 4. Kaplan-Meier curve of severe morbidity probability by MV2 allocation not censoring at OPV campaigns.** Severe morbidity rate ratios (SMRRs) were estimated from Cox proportional hazards models with time since enrolment as the underlying time variable and observations were censored at migration, 48 months of age or first day of national MV campaign. Hospital admissions were analysed as recurrent events, and hospital admissions >=1 day from the latest discharge counted as a new event. Hospital admissions that resulted in death were counted as a singular event. Abbreviations: MV2, Second measles vaccine.


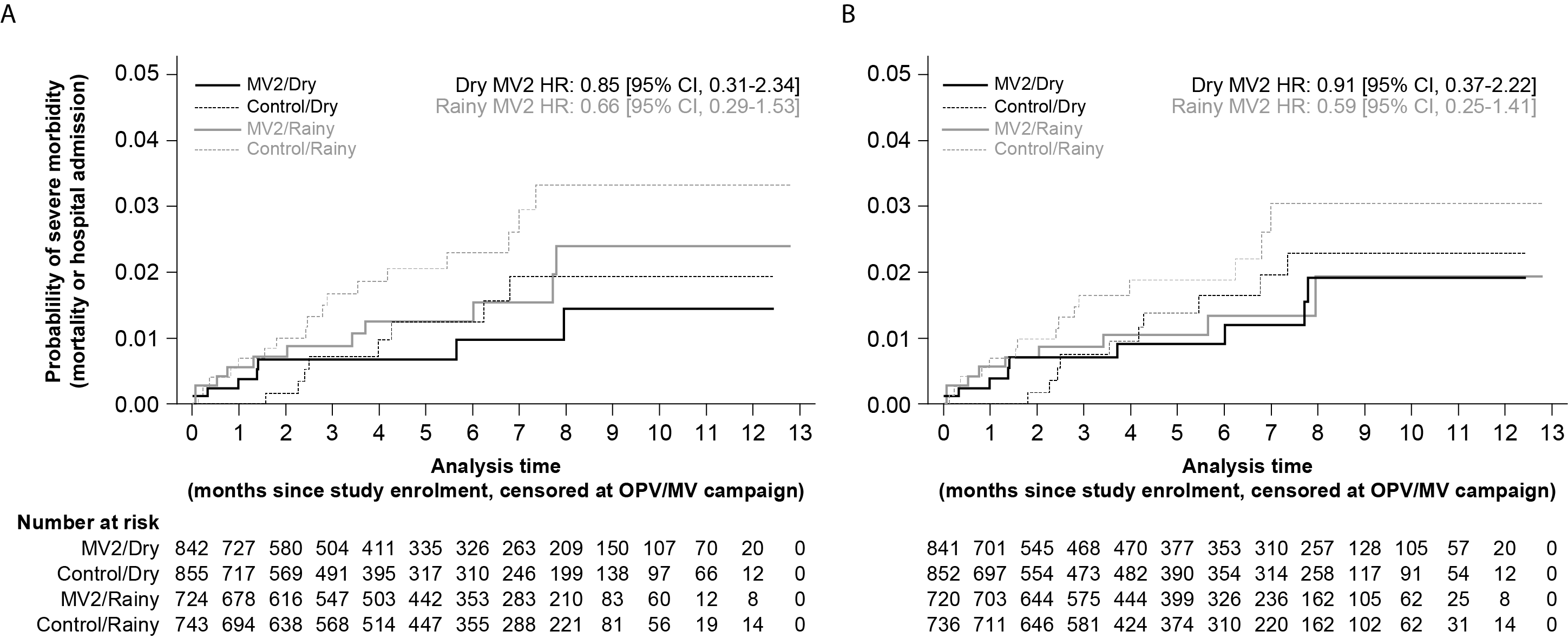


**Supplementary Figure 5. Kaplan-Meier curve of seasonal influences on severe morbidity probability by MV2 allocation.** Severe morbidity rate ratio (SMRR) was estimated from a Cox proportional hazards model including an interaction term between allocation and either season of enrolment (Dry season: 1 Dec - 31 May, Rainy season: 1 Jun - 30 Nov) (A) or season at risk (B) with time since enrolment as the underlying time variable and observations were censored at migration, 48 months of age or first day of national OPV or MV campaign. Hospital admissions were analysed as recurrent events, and hospital admissions >=1 day from the latest discharge counted as a new event. Hospital admissions that resulted in death were counted as a singular event. Abbreviations: MV2, Second measles vaccine.

**Supplementary Table 1. Characteristics of included and excluded children**

|  | **Included**  **N=3,164** | **Excluded**  **N=3,595^b^** | **P value^c^** |
| --- | --- | --- | --- |
| Age, median (25p-75p) | 590 (539-686) | 536 (534-541)^a^ | <0.001 |
| Male sex | 50% (1,588) | 52% (1,864)^a^ | 0.16 |
| Twin/triplet^a^ | 4.1% (131) | 3.4% (122) | 0.11 |
| Ethnicity^a^ |  |  | <0.001 |
| Pepel | 26% (821) | 21% (761) |  |
| Fula | 20% (637) | 39% (1,379) |  |
| Other | 54% (1,692) | 40% (1,441) |  |
| District |  |  | <0.001 |
| Bandim | 44% (1,382) | 51% (1,848) |  |
| Belem/Mindara | 15% (472) | 15% (523) |  |
| Cuntum | 41% (1,309) | 34% (1,223) |  |
| Results are presented as median (25p – 75p) for continuous variables and as percentages (n) for categorical variables. ^a^Different n due to missing values, which were <0.5% except for age among excluded children (number of missing = 2,372). Since children that were not eligible were not visited, there was no date at which to calculate the age. ^b^Excluded children before and after randomization, except program errors (n=305), not visited due to insufficient time (n=45) and not contributing time-at-risk (n=9). ^c^Wilcoxon ranked sum test for continuous variables, and χ2 test for categorical variables. The Fula ethnicity, which are relatively common among the Bandim district, have the poorest adherence to the vaccination schedule and were therefore more likely to not be eligible for inclusion. | | | |

**Supplementary Table 2. Number of missing values in baseline characteristics**

| **Characteristic** | **MV2 (n = 1,566)** | **Control (n = 1,598)** |
| --- | --- | --- |
| **Child factors** |  |  |
| Anthropometrics |  |  |
| Weight in kilograms | 0.1% (1) | 0.1% (1) |
| Height in cm | 0.3% (5) | 0.4% (7) |
| MUAC in mm | 0.1% (1) | 0.1% (1) |
| Temp in °C | 0.1% (2) | 0.2% (3) |
| BCG scar | 0.2% (3) | 0.4% (7) |
| Symptoms |  |  |
| Any symptom | 0.1% (2) | 0.3% (4) |
| Congestion/Rhinorrhea | 0.1% (2) | 0.1% (1) |
| Cough | 0.2% (3) | 0.3% (4) |
| Fever | 0.2% (3) | 0.3% (4) |
| Vomiting | 0 | 0.1% (1) |
| Diarrhea | 0 | 0.1% (2) |
| Breathing difficulties | 0.1% (2) | 0.3% (4) |
| Convulsions | 0.3% (4) | 0.4% (6) |
| Medication use |  |  |
| Any | 0.2% (3) | 0.1% (2) |
| Paracetamol | 0.2% (3) | 0.2% (3) |
| Antimalarials | 1.0% (15) | 0.9% (15) |
| Antibiotics | 0.4% (6) | 0.5% (8) |
| Still breastfed | 0.3% (5) | 0.3% (5) |
| Hospital admission before enrolment | 0.1% (1) | 0.5% (8) |
| Child has had measles | 0.2% (3) | 0.1% (1) |
| **Socio-economic factors** |  |  |
| Zinc roofing material | 0.6% (10) | 0.8% (12) |
| Functioning electricity | 0.3% (4) | 0.8% (12) |
| Indoor toilet | 0.9% (14) | 1.0% (16) |
| Number of persons per room | 0.3% (4) | 0.3% (4) |
| Number of persons per bed | 0.6% (9) | 0.4% (7) |
| Sleeping under bed net | 1.0% (16) | 0.8% (12) |
| Antimalarials in the household | 2.2% (34) | 1.5% (24) |
| Pigs in the household | 0.5% (8) | 0.8% (12) |
| Measles in the household | 1.9% (30) | 1.8% (29) |
| **Maternal factors^a^** |  |  |
| Maternal age in years | 1.5% (24) | 1.8% (28) |
| Maternal schooling | 2.6% (40) | 2.2% (35) |
| Maternal MUAC | 10.0% (156) | 10.8% (172) |
| Maternal measles infection | 8.8% (138) | 8.4% (134) |
| Maternal measles vaccination | 4.7% (73) | 5.4% (86) |
| Maternal BCG scar | 10.7% (167) | 10.8% (173) |
| ^a^Numbers in maternal factors are relatively high as the mothers needed to be present at the home visit or enrolment (MUAC and BCG scar) to retrieve the information | | |

**Supplementary Table 3. Mortality and hospital admissions by MV2 allocation**

|  | Mortality rate  [Deaths/100 Person years] (n) | | MRR (95% CI) (MV2/control) |
| --- | --- | --- | --- |
|  | MV2 | control |  |
| Mortality | 0.1 [1/6.9] (1,566) | 0.3 [2/6.9] (1,598) | 0.50 (0.04-5.46)^a^ |
|  | Hospitalization rate  [Hospital admissions/100 Person years] (n) | | HRR (95% CI) (MV2/control) |
|  | MV2 | control |  |
| Hospital admissions | 2.5 [17/6.9] (1,566) | 3.5 [24/6.9] (1,598) | 0.71 (0.36-1.38)^b^ |
| Mortality rate ratio (MRR) and hospitalization rate ratio (HRR) were estimated from a Cox proportional hazards model with time since enrolment as the underlying time variable and observations were censored at migration, 48 months of age or first day of national OPV or MV campaign. Hospital admissions were analysed as recurrent events, and hospital admissions >=1 day from the latest discharge counted as a new event. ^a^Proportional hazards test, p=0.19. ^b^Proportional hazards test, p=0.39. Abbreviations: MV2, Second measles vaccine. | | | |

**Supplementary Table 4. Seasonal influences on severe morbidity by MV2 allocation**

|  | Severe morbidity rate  [Deaths or Hospital admissions/100 Person years] (n) | | SMRR (95% CI) (MV2/control) |
| --- | --- | --- | --- |
|  | MV2 | control |  |
| **Season of enrolment** | | | |
| Dry season | 2.0 [7/3.4] (842) | 2.4 [8/3.3] (855) | 0.85 (0.31-2.34)^a^ |
| Rainy season | 3.2 [11/3.5] (724) | 4.8 [17/3.5] (743) | 0.66 (0.29-1.53)^a^ |
| **Season at risk^a^** | | | |
| Dry season | 2.6 (9/3.5] (1257) | 2.8 [10/3.5] (1276) | 0.91 (0.37-2.22)^b^ |
| Rainy season | 2.7 (9/3.4] (1057) | 4.5 (15/3.3] (1058) | 0.59 (0.25-1.41)^b^ |
| Severe morbidity rate ratios (SMRR) were estimated from a Cox proportional hazards model including an interaction term between allocation and either season of enrolment or season at risk (Dry season: 1 Dec - 31 May, Rainy season: 1 Jun - 30 Nov) with time since enrolment as the underlying time variable and observations were censored at migration, 48 months of age or first day of national OPV or MV campaign. Hospital admissions were analysed as recurrent events, and hospital admissions >=1 day from the latest discharge counted as a new event. Hospital admissions that resulted in death were counted as a singular event. ^a^Individuals can contribute to both dry and rainy season depending on time of enrolment and follow-up duration. ^b^Proportional hazards test, p=0.75. ^c^Proportional hazards test, p=0.62. Abbreviations: MV2, Second measles vaccine. | | | |

**Supplementary Table 5. Consultations in first 14 days by MV2 allocation**

|  | Consultation rate  [Consultations/100 Person years] (n) | | CRR (95% CI) (MV2/control) |
| --- | --- | --- | --- |
|  | MV2 | control |  |
| First 14 days after enrolment | 185.7 [109/0.6] (1566) | 186.6 [111/0.6] (1598) | 1.00 (0.76-1.30)^a^ |
| Consultation rate ratio (CRR) was estimated from a Cox proportional hazards model with time since enrolment as the underlying time variable and observations were censored at migration, 48 months of age or first day of national OPV or MV campaign. Only the first 14 days (MV adverse events window) were included in this analysis. Consultations were analysed as recurrent events, and consultations >=1 day from the latest consultation counted as a new event. ^a^Proportional hazards test, p=0.97. Abbreviations: MV2, Second measles vaccine. | | | |
